# Commentary: Enhanced Interplay of Neuronal Coherence and Coupling in the Dying Human Brain

**DOI:** 10.3389/fnagi.2022.899491

**Published:** 2022-05-18

**Authors:** Bruce Greyson, Pim van Lommel, Peter Fenwick

**Affiliations:** ^1^Department of Psychiatry & Neurobehavioral Sciences, University of Virginia School of Medicine, Charlottesville, VA, United States; ^2^Department of Cardiology, Rijnstate Hospital, Arnhem, Netherlands; ^3^Department of Psychiatry, Emeritus Maudsley Hospital and Emeritus Kings College Institute of Psychiatry, London, England

**Keywords:** near-death, end-of-life, life recall, coherence, coupling

## Introduction

We read with great interest in recent article by Vicente et al. (2022) that described continuous electroencephalography (EEG) recording in an 87-year-old patient who unexpectedly suffered a cardiac arrest after traumatic bilateral subdural hematoma and status epilepticus. The authors reported that brainwaves in the delta, beta, alpha, and gamma frequency ranges were decreased, but a higher percentage of relative gamma power was observed compared to the interictal interval, and cross-frequency coupling revealed modulation of left-hemispheric gamma activity by alpha and theta rhythms.

We greatly appreciated the authors' caution in writing that, “Given that cross-coupling between alpha and gamma activity is involved in cognitive processes and memory recall in healthy subjects, it is intriguing to speculate that such activity could support a last ‘recall of life’ that may take place in the near-death state” (2022, p. 9). We also appreciate their acknowledging that the lack of any normal brain electrical activity recorded from the patient that could serve as a baseline for comparison cast doubts on any interpretation of the findings (2022, p. 9). However, while we agree that this case report is intriguing, we wish to raise some questions about the interpretation and the implications of their data.

## Gamma Oscillations in the Dying Process

The authors reported that there was a temporary increase in gamma power when bilateral hemispheric activity ceased, but that it declined after what they described as cardiac arrest. In other words, the EEG recorded from this patient did not show an increase in absolute gamma activity after cardiac arrest, but rather showed a reduction in absolute gamma waves. It was only the relative amount of gamma that was increased compared to alpha, beta, and delta.

## Source of Gamma Oscillations

Vicente et al. (2022) noted that increased gamma power and long-range gamma synchronization have been identified in conscious perception; but they have also been found across the neocortex in association with a wide variety of brain circumstances (Muthukumaraswamy, 2013) ranging from ongoing tonic pain (Schulz et al., 2015) to preparation for and execution of movements (Ulloa, 2022). Vicente et al. discerningly listed several reasons not to place too much importance on this one patient's EEG: the patient's traumatic brain injury and subdural hematoma, the anesthesia-induced loss of consciousness, the dissociative drugs given to the patient, the anticonvulsant drugs to control his seizures, and the patient's asphyxia and hypercapnia. These confounding variables raise questions about the interpretation of the relative increase in gamma oscillations seen following cardiac arrest in this patient.

There is additional uncertainty about whether the gamma waves recorded in the EEG entirely reflected brain activity or were at least in part measuring muscle contractions. Contamination of EEG recordings by muscle artifact is a well-recognized problem, especially in the high-frequency gamma range, leading to erroneous estimates of EEG spectral power and coherence (Goncharova et al., 2003; Pope et al., 2009; Fitzgibbon et al., 2013). The peak power of the gamma oscillations recorded from this patient between burst suppression and cardiac arrest was in the upper gamma range typical of muscle activity, and it occurred primarily on the frontal and temporal electrodes, where muscle artifact is most often found. This raises questions as to whether the gamma oscillations reflected neuronal activity or frontalis and temporalis muscle activity. Unfortunately, the use of the global EEG output in this study to calculate the relative and absolute power of the frequency bands obscures any spatial information about the source of those electrical signals (Muthukumaraswamy, 2013).

## Determination of Cardiac Arrest

There are also questions about the identification of cardiac arrest in this study. The authors defined cardiac arrest as “the abrupt loss of heart function measured by the inability to obtain pulse activity in the ECG [electrocardiogram]” (p. 2–3). According to worldwide accepted cardiological criteria, cardiac arrest is caused by ventricular fibrillation (VF) or asystole. This patient developed ventricular tachycardia with apneustic respirations and a clinical cardiorespiratory arrest; Figure 2A in the article by Vicente et al. (2022) showed the patient having ventricular tachycardia but not VF or asystole, and showed continued ECG activity past the moment marked in the figure as the time of the cardiac arrest. Thus, at the time of the EEG changes, the patient had not in fact experienced cardiac arrest but was still having ECG activity.

## Discussion

The case presented by Vicente et al. (2022) should be viewed in the context of decades of clinical experience and research suggesting that brain activity decreases after about 8 s after the onset of cardiac arrest, and becomes a flatline EEG after 18 seconds (Clute and Levy, 1990; Losasso et al., 1992; de Vries et al., 1998; van Lommel, 2011). Reports of cases in which the EEG was monitored during cardiac arrest, for example during surgery with EEG monitoring, suggest that the EEG flatlines after an average of 15 s and remains flat despite external resuscitation (Hossmann and Kleihues, 1973; Moss and Rockoff, 1980; Clute and Levy, 1990; Losasso et al., 1992). Progression to a flatline EEG always occurs in these prior studies within 10–20 s from the onset of cardiac arrest (Clute and Levy, 1990; Losasso et al., 1992; de Vries et al., 1998; Parnia and Fenwick, 2002), and the EEG remains flat during the cardiac arrest until cardiac output has been restored by defibrillation (Fischer and Hossmann, 1996; Marshall et al., 2001).

In a prospective research study, Norton et al. (2017) monitored continuous EEG and cardiac function in four intensive care unit (ICU) patients after life-sustaining therapy was withdrawn. Three of those four patients showed EEG inactivity (defined as amplitude of <2 μV, following recommended guidelines for EEG testing in brain death) prior to the cessation of arterial blood pressure and ECG activity; the fourth patient showed infrequent single delta bursts for more than 10 min after electrocardiographic cessation.

More recently, Matory et al. (2021) studied continuous EEG recording from 19 ICU patients who died from heart attacks despite receiving treatment. Two of the 19 had some brief EEG activity (for up to 2 min) following the last QRS complex recorded in the ECG, and 11 showed EEG activity briefly (for <5 min) after blood flow to the brain had ceased, as estimated by blood pressure and heart rate falling below set thresholds. After blood flow to the brain had ceased, full-spectrum log power and coherence both decreased on EEG, whereas delta and theta permutation entropy increased.

In summary, we agree with Vicente et al. (2022) that the case they described is intriguing enough to stimulate speculation, and we believe it warrants further into brain function throughout the terminal state.

## Author Contributions

BG and PL wrote the manuscript. All authors contributed to the article, approved the final version, and agree to be accountable for the content of the work.

## Conflict of Interest

The authors declare that the research was conducted in the absence of any commercial or financial relationships that could be construed as a potential conflict of interest.

## Publisher's Note

All claims expressed in this article are solely those of the authors and do not necessarily represent those of their affiliated organizations, or those of the publisher, the editors and the reviewers. Any product that may be evaluated in this article, or claim that may be made by its manufacturer, is not guaranteed or endorsed by the publisher.
